# Supplementary material for: The potential of mycelium from mushroom-producing fungi in alternative protein production: a focus on fungal growth, metabolism, and nutrition
Source: Curr Res Food Sci. 2025 Dec 15;12:101278. doi: 10.1016/j.crfs.2025.101278 (PMC12810349; doi:10.1016/j.crfs.2025.101278)

The potential of mycelium from mushroom-producing fungi in alternative protein production:

a focus on fungal growth, metabolism, and nutrition

**Authors:** Jasper Zwinkels^a^, Stef van Oorschot^a^, Oscar van Mastrigt^a^, Eddy J. Smid^a,1^

^a^ Food Microbiology, Wageningen University, Wageningen, the Netherlands

^1^Corresponding author, eddy.smid@wur.nl

# Supplementary material

**Supplementary figure 1 |** Cross-sectional appearance of brown rice, brewer's spent grain (BSG) and lupin fermented with *S. rugosoannulata*, *S. commune, P. cinnabarinus*, *V. volvacea*, *P. pulmonarius* and *R. microsporus*.


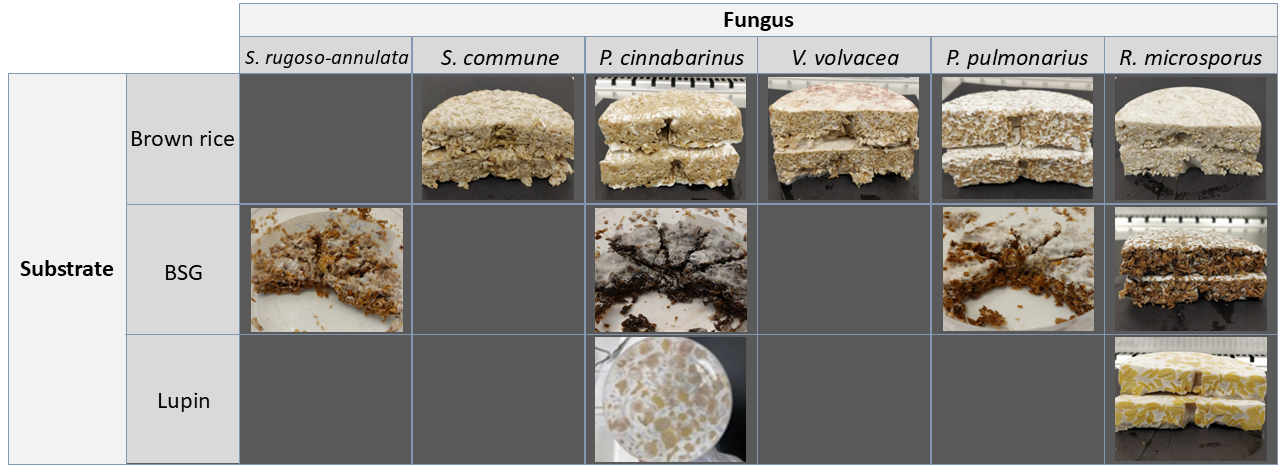


| **Supplementary figure 2 \|** Temperature profile of Brewer’s spent grain fermented by *P. pulmonarius* at different maltose concentration (A; 0-10%) or pH values (B;3.5-6.5). |
| --- |
| B |

**Supplementary figure 3 |** Ergosterol (A) and glucosamine (B) concentration (g/100 g DW) in pure mycelium of *S. rugosoannulata* (red), *S. commune* (orange), *P. cinnabarinus* (green), *V. volvacea* (turquoise), *P. pulmonarius* (blue) or *R. microsporus* (pink).


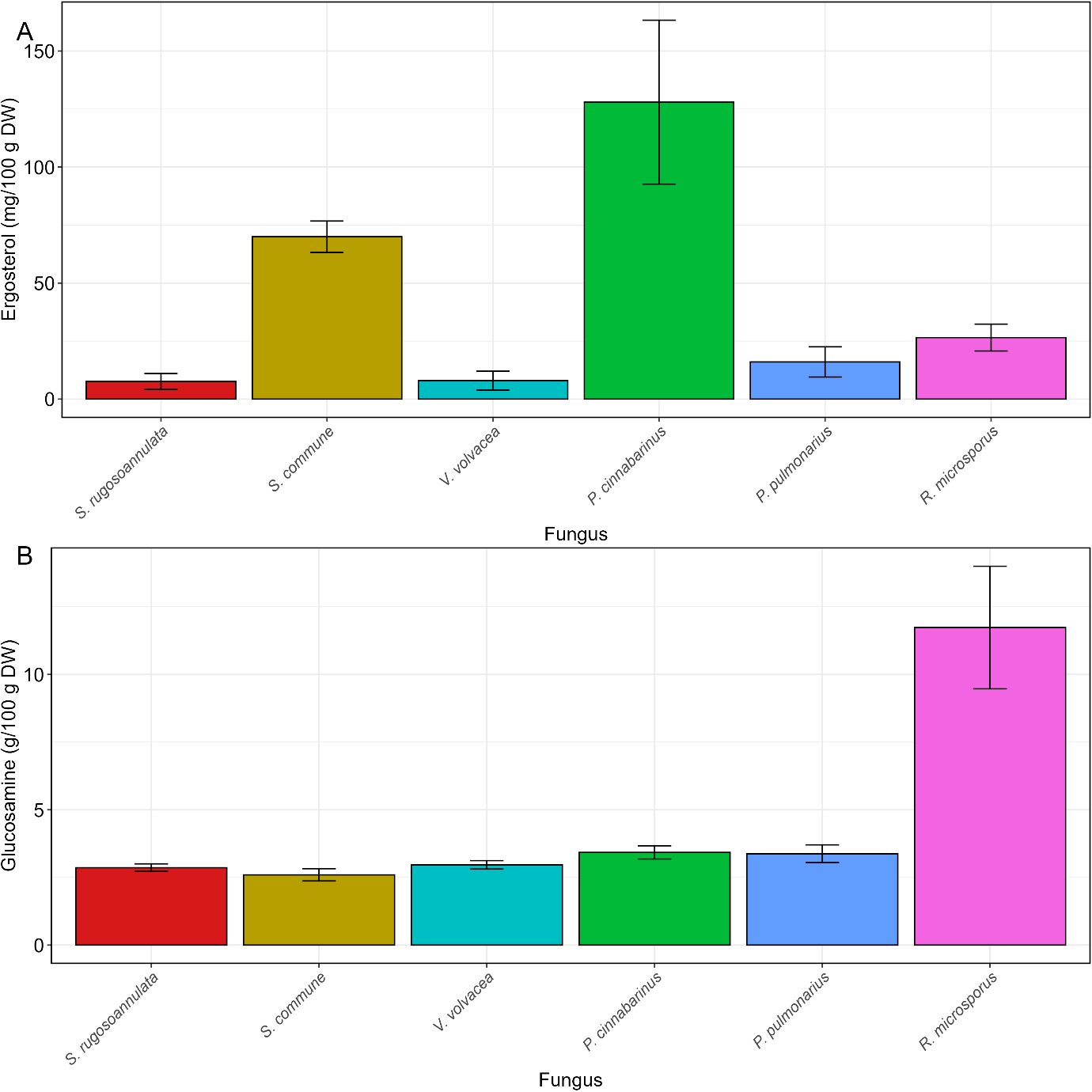


**Supplementary figure 4 |** Correlation between fungal biomass based on glucosamine and ergosterol (g/100 g DW) in *R. microsporus* fermentations. R2 and significance in the top left corner.


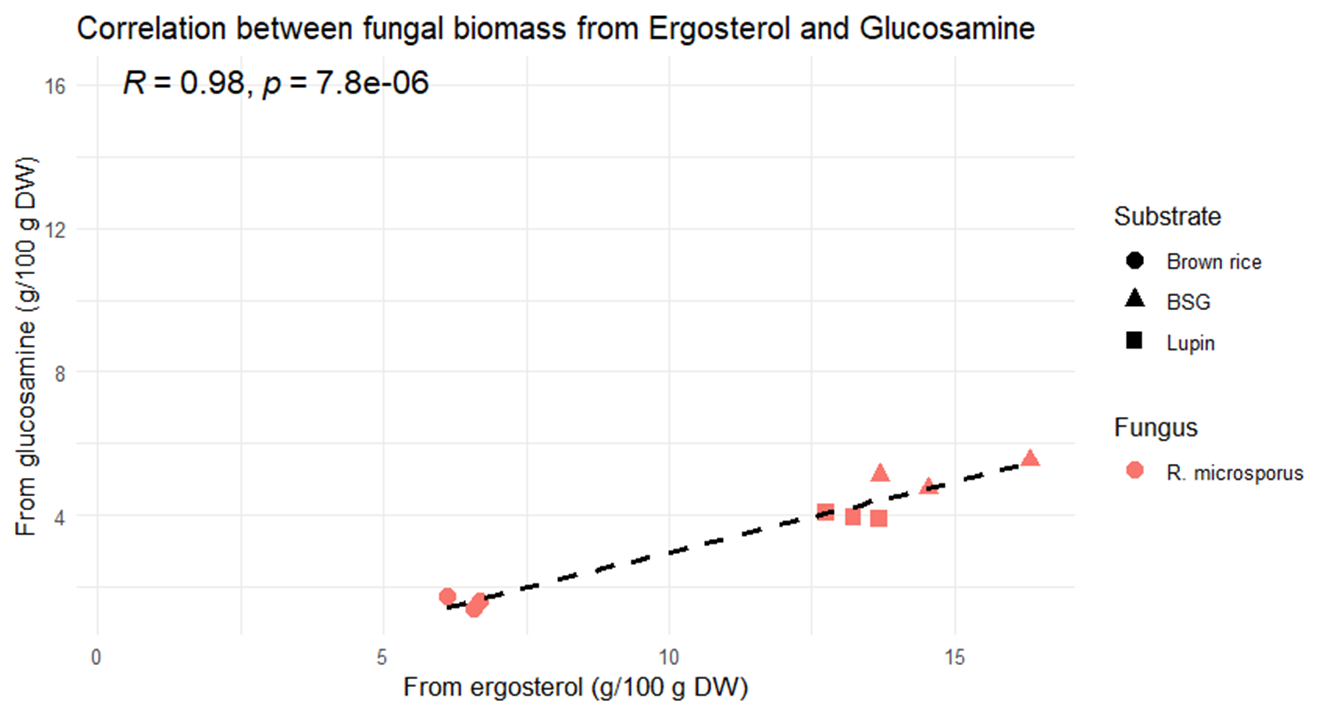

Supplement: Multimedia component 1 [file mmc1.docx]
